# A Bio-Inspired Learning Dendritic Motion Detection Framework with Direction-Selective Horizontal Cells

**DOI:** 10.3390/biomimetics10050286

**Published:** 2025-05-02

**Authors:** Tianqi Chen, Yuki Todo, Zhiyu Qiu, Yuxiao Hua, Hiroki Sugiura, Zheng Tang

**Affiliations:** 1 Division of Electrical Engineering and Computer Science, Kanazawa University, Kanazawa 920-1192, Japan; chentianqi@stu.kanazawa-u.ac.jp (T.C.); qiuzy1916@stu.kanazawa-u.ac.jp (Z.Q.); sh13818971028@gmail.com (Y.H.); sugiurahiroki@stu.kanazawa-u.ac.jp (H.S.); 2 Faculty of Electrical, Information and Communication Engineering, Kanazawa University, Kanazawa 920-1192, Japan; 3 Institute of AI for Industries, Chinese Academy of Sciences Nanjing, Nanjing 210008, China

**Keywords:** motion direction detection, artificial visual system, dendritic neuron model, synaptic learning, machine learning, bio-inspired model, noise robustness, bio-inspired computing

## Abstract

Motion direction detection is an essential task for both computer vision and neuroscience. Inspired by the biological theory of the human visual system, we proposed a learnable horizontal-cell-based dendritic neuron model (HCdM) that captures motion direction with high efficiency while remaining highly robust. Unlike present deep learning models, which rely on extension of computation and extraction of global features, the HCdM mimics the localized processing of dendritic neurons, enabling efficient motion feature integration. Through synaptic learning that prunes unnecessary parts, our model maintains high accuracy in noised images, particularly against salt-and-pepper noise. Experimental results show that the HCdM reached over 99.5% test accuracy, maintained robust performance under 10% salt-and-pepper noise, and achieved cross-dataset generalization exceeding 80% in certain conditions. Comparisons with state-of-the-art (SOTA) models like vision transformers (ViTs) and convolutional neural networks (CNNs) demonstrate the HCdM’s robustness and efficiency. Additionally, in contrast to previous artificial visual systems (AVSs), our findings suggest that lateral geniculate nucleus (LGN) structures, though present in biological vision, may not be essential for motion direction detection. This insight provides a new direction for bio-inspired computational models. Future research will focus on hybridizing the HCdM with SOTA models that perform well on complex visual scenes to enhance its adaptability.

## 1. Introduction

The visual system is the primary source of information resources for humans, with studies proving that over 80% of sensory inputs are directly or indirectly based on vision [1,2]. It is a highly efficient mechanism with the capability of processing multi-channel inputs, allowing humans to understand complex environments and serving as a fundamental basis for behavior [3,4,5]. Within this system, photoreceptors, specifically rods cells and cones cells, capture light and transpose it into signals in neurons [6]. Cone cells, in particular, play a crucial role in color vision by processing signals from three channels that are divided according to the wavelength, corresponding to red, green, and blue (RGB) inputs [7,8]. The principles of multi-channel visual processing have inspired modern artificial neural network models [9,10,11]. Convolutional neural networks (CNNs) and vision transformers (ViTs) have been developed to handle preprocessed motion direction detection tasks in some situations. CNNs utilize spatial hierarchies to extract motion features, while ViTs employ self-attention mechanisms to capture long-range dependencies, leading to superior performance in image classification and recognition tasks [12,13,14]. However, despite these advancements, many artificial models struggle to replicate the full capabilities of biological vision. Models based on artificial neural networks are designed to process RGB images, though they may still fail to detect pixel-level motion or generalize to diverse image transformations [15,16]. This limitation underscores the need for more adaptive solutions that are more likely to be bio-inspired by the visual systems. Many functions of the visual system are shaped by learning and environmental factors, highlighting the potential of bio-inspired systems to replicate these capabilities [17,18].

The retina is mostly regarded as the first processing terminal of the visual system [2,7]. It integrates and modulates visual input through multiple neural layers [19,20]. This suggests that bio-inspired models or neural networks based on retinal mechanisms have the potential to surpass traditional neural network models in design. The direction selectivity is a very important function in the retina of mammals; this function helps mammals to enable detecting ability and motion direction processing [18,21]. These task of direction selectivity solve such situations as navigation and predator avoidance, which depend on the behavior of them. Hubel and Wiesel’s pioneering work in the 1960s revealed that neurons in the primary visual cortex (V1) are selectively responsive to specific orientations and directions of motion [22]. The model they proposed suggests that the alignment of the structures in the retina contributes to the selectivity in the primary cortex. In their further research, it was found that the simple cells and complex cells are located in the V1 of the primary cortex. These kinds of cells have the ability of direction selection when groups of light spots or thin lines are detected, which shows that the selectivity is widely spread in the retina [23].

Horizontal cells (HCs), which are necessary for early-stage signal processing, are also included in the multiple neural layers of the retina [24,25,26]. HCs are known to mediate lateral inhibition, enhancing contrast and refining visual perception [27,28]. Recent studies suggest that horizontal cells may also play a critical role in determining direction selectivity by influencing the ganglion cells within the retina [29]. Specifically, research has shown that injecting a current into horizontal cells affects the activity of the retinal ganglion cells involved in detecting both fast and slow motion, as well as direction selectivity [30,31]. This result shows that HCs may contribute significantly to the modulation of direction-selective responses in the retina. Another work of research on retina cells showed that the direction-selective retinal ganglion cells integrate global motion, generating sparse yet consistent responses aligned with specific motion directions, especially in large data populations. Studies have demonstrated that local motion decoders rely on correlated neural activity to achieve spatial resolution, whereas global motion decoders assume independent inputs to facilitate continuous motion direction readout [32,33]. This highlights the critical role of the ganglion cells in global motion perception. These processed signals are subsequently transmitted to the lateral geniculate nucleus (LGN) and deeper cortical areas for further interpretation, forming the foundation for biological decision making [19,34]. Compared to traditional neural-network-based methods, bio-inspired models offer faster processing speeds and higher accuracy, particularly in tasks closely related to human visual perception [11,35]. Traditional neural networks, which rely on perceptron-based architectures, often lose critical associations between input signals [36,37]. While transformer-based attention mechanisms provide a potential solution, they require extensive computational resources and significant processing time [14,38].

Dendritic structures are fundamental to neuronal processing, particularly in the visual system. As a key function of the brain—an organ composed of over $10^{11}$ neurons—the visual system relies on the intricate interactions between these neurons, which are mediated by their dendritic structures. Remarkably, this system facilitates more than $10^{15}$ interactions, highlighting the critical role of dendritic organization in neural computation [39,40]. Unlike perceptron-based models, which disregard the complex interactions of synapses and dendrites, dendritic neuron models integrate the excitatory and inhibitory inputs across spatial locations, enabling nonlinear signal processing in model [41,42]. This enhances the ability to capture local features and reconstruct the importance of individual inputs in classification tasks. Dendritic neuron models provide an effective framework for input information processing by mimicking the intricate interactions of local signals, which not only improves the computational and learning efficiency but also the generalization capability and robustness [42,43].

Although dendritic-neuron-based learning models show promise, some dendritic neuron models remain limited to grayscale image processing and lack the ability to handle multi-channel inputs such as RGB images [44]. Consequently, they fail to capture the full complexity of natural vision. While certain models have explored multi-channel processing, their structures remain unclear due to the deep and complex processing mechanisms of the retina in the LGN [19,45]. This lack of clarity makes conclusive model development challenging and increases architectural complexity. As a result, multi-channel processing based on the LGN may lack sufficient biological interpretability [46]. These limitations suggest that such models may not fully replicate the biological efficiency and adaptability required for complex visual tasks.

Based on these principles, our previous study introduced an improved artificial visual system (AVS) that uses dendritic-neuron-based structures to replicate how the retina processes information. Unlike earlier single-channel systems, our model can detect motion direction in RGB images instead of grayscale ones. By leveraging the direction selectivity of horizontal cells, we improve motion detection, making the approach more aligned with how biological vision works [47]. This allows the system to effectively process multi-channel visual signals, combining color information and motion direction in a way that closely resembles natural vision. As a result, our model brings artificial vision closer to the complexity and functionality of biological vision.

Despite the advancements in visual motion detection models, an essential scientific challenge appears. The challenge is to replicate the direction selectivity and noise robustness of a biological-visual-system-based computational model, especially for the pixel-level motion scenario. While deep learning models perform well at high-level motion representation, they often struggle with frame-by-frame processing and lead to high computational costs. In contrast, our previous model, AVS, exhibits a higher computational efficiency but relies on unclear biological mechanisms such as the LGN with its multiplicative operations hypothesis. To address these limitations, we propose a learnable horizontal-cell-based dendritic neuron model (HCdM), which utilizes the established direction selectivity of HCs and a mechanism with biological evidence rather than the LGN, which locates at deeper structure in visual system. The proposed model not only enhances the biological reasonability but also the computational efficiency compared to other models. Thus, the HCdM has a balance with appropriate reduction in computational cost and improvement of reasonability in motion direction tasks.

The novelty of this work mainly lies in its bio-inspired dendritic structural connection to HCs, which replicates localized visual computation and enables retinal direction selectivity, yet such features are often overlooked. Although most computational models ignore this biological mechanism, it plays a crucial role in recognizing object edges from background noise. This structure enables the HCdM to not only process RGB motion direction with high precision but also maintain robustness under noisy conditions and dataset shifts. Our goal is to explore whether a hierarchical dendritic framework with synaptic learning can simulate the learning process of direction selectivity and outperform conventional deep models under constrained scenarios.

After simulations of our model, the experimental results indicate that our model accurately identifies nearly all motion directions in pixel-level object movement across eight directions in consecutive frames. In comparison with conventional CNNs, which lack multi-channel and multi-modal processing capabilities, we extended the CNNs through specific methods by dataset preprocessing to incorporate them into our comparisons. For models with multi-modal processing abilities, such as ViTs, we employed both a spatiotemporal ViT (stViT) and a two-stream ViT (2sViT). The former expands along the temporal sequence for motion direction detection, while the latter defines the dataset as multi-modal and processes it accordingly [48,49]. Self-supervised temporal learning models such as DuTriNet, which incorporates a flow-intensity-based non-parametric frame sampling algorithm within a triplet Siamese architecture, may be considered as solutions to motion direction detection models due to their strong ability to identify significant motion shifts, such as body pose transitions in action recognition tasks [50]. However, these models lack sensitivity to the subtle, pixel-level displacements present in our dataset, which is the most crucial difference from our testing dataset to its aiming dataset. These methods helps models finish the recognition tasks on more than a single image. Experimental results demonstrate that our model exhibits high accuracy and computational efficiency. Simulations further confirm its precision, even when trained on relatively small training datasets, which is an advantage that is not observed in other models. This study simulates the learning process of direction selectivity in the brain, reinforcing its basic biological theory. Additionally, our simulations suggest that, in RGB images, horizontal cells theoretically achieve direction selectivity through their relative influence on other cells. This supports the role of computational simulations in exploring deeper principles in biological theories.

Our study draws inspiration from neuroscience, particularly the work of Hubel and Wiesel, to explore how dendritic structures contribute to direction selectivity in visual processing. By integrating this principle into our model, we not only improve its ability to learn direction selectivity but also enhance its biological plausibility. Theoretically, our model’s dendritic structure that amplifies imperceptible motion cues between frames enables the detection of extremely fine-grained changes. This approach strengthens the alignment of our model with biological mechanisms, making it a more accurate representation of how biological vision systems operate. Moreover, our approach has wide-ranging potential applications in motion direction tasks, such as autonomous driving, robotic vision, and medical imaging analysis. Practically, this makes our model particularly suitable for pixel-level motion tasks like precision manufacturing or cell movement tracking, where detecting subtle displacements is crucial. These examples demonstrate the practical value of our findings and underscore the broader impact of bio-mimetic computational models in solving real-world problems.

## 2. Methods

To simulate motion direction detection process in biological visual system, we designed our model following the functional structure of the retina. We began with a layerwise model which replicated the key components of retina. First, we simulated the outer nuclear cell layer where photoreceptor cells receive light input and separated it into three channels. Next, we modeled the inner nuclear layer, focusing on the HCs and their lateral inhibitory functions that modulate bipolar cell outputs to enable contrast enhancement and motion direction selectivity. We employed dendritic neuron structures to integrate the information features in motion direction detection in the ganglion cell layer, where these signals are then passed to. Finally, we implemented a backpropagation-based training mechanism that adjusts the connection positions of the synapses according to the prediction errors. This structure enables the model to not only reflect biological processing principles but also learn motion direction efficiently in RGB images.

### 2.1. Retinal RGB Image Processing

**Figure 1:**
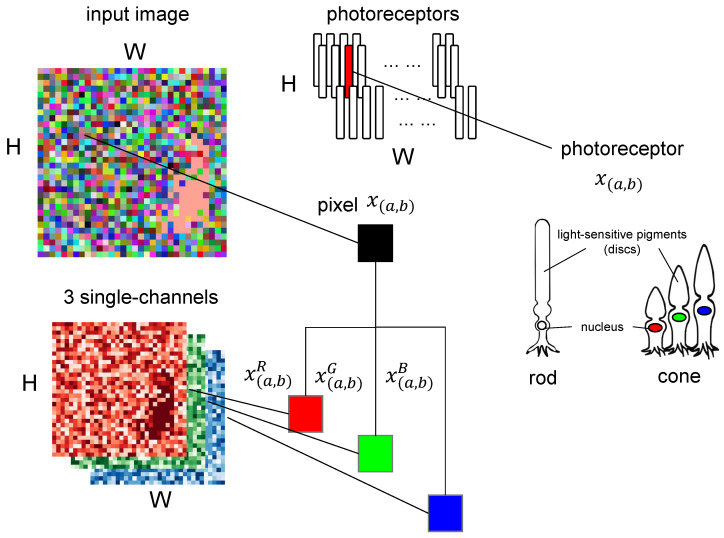
This figure shows the input image processed by the rod cells and cone cells in photoreceptors with wavelength-based light channel.

In retina, three types of cone cells are considered to process color information. Each type of cone cell is sensitive to its own responsible wavelength range of light. This biological mechanism, based on the detection of three distinct wavelengths, closely aligns with the RGB color model used in digital imaging, as the RGB color spectrum also represents different wavelength ranges of light [51]. As shown in Figure 1, cone cells enable color perception, while rod cells support low-light vision by detecting the presence or absence of light [52]. In the figure, we assume that the input image is made up of H×W pixels and active H×W photorecpetor cells; a single-pixel $x_{\left(a,b\right)}$ (black square) in the image is composed of three channel lights with different wavelengths that are received by the cone cells of different channels: $x_{\left(a,b\right)}^{R}$, $x_{\left(a,b\right)}^{G}$, and $x_{\left(a,b\right)}^{B}$. The similarity between biological color perception and the RGB format provides a strong basis for using three-channel image datasets in computational artificial visual systems [53].

Furthermore, the retina processes color information in parallel, where different color channels are handled independently before being integrated at higher levels of processing [54]. This parallel processing suggests that an artificial visual system should also adopt a multi-channel approach to better simulate biological vision [55]. By maintaining separate processing pathways for each channel, our model is designed to capture motion and spatial information as a ‘feature’ in a biological mechanism.

### 2.2. On–Off Response Mechanism Based on Horizontal Cells

**Figure 2:**
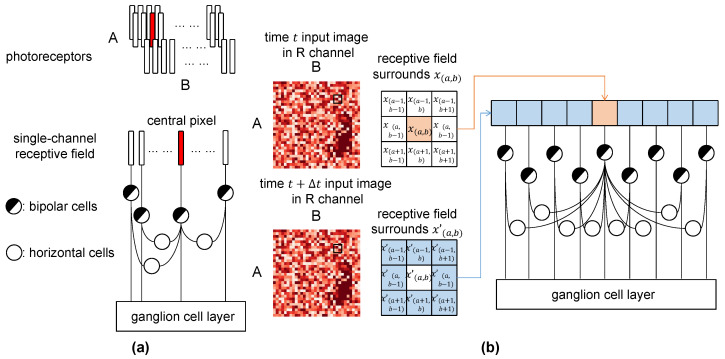
In the figure, (**a**) illustrates the biological positioning of horizontal cells in the retina, and (**b**) shows how local receptive fields contribute to direction selectivity through pixel comparisons in a single RGB channel.

The detection of motion direction begins when the retina receives light stimuli [31]. Photoreceptor cells convert these stimuli into electrical signals, which are processed by various neural layers in the retina, including horizontal cells [28]. Horizontal cells play a crucial role in lateral inhibition in the process; the HCs adjust the response of bipolar cells and contribute to direction selectivity [26]. As shown in Figure 2a, HCs are located in the retina between photoreceptors and bipolar cells, modulating visual signals before they reach the ganglion cells [24].

In the case of RGB image processing, our model extends the horizontal cell mechanism to handle the complexity introduced by multi-channel inputs [27]. Each color channel is processed separately, mimicking how biological vision treats different wavelengths of light [25]. For each channel, photoreceptors transform incoming light from different channels of cone cells into signals representing pixel intensity [28]. These signals are then processed by HCs to detect local intensity variations, thereby enabling motion detection by recognizing the object boundary [30].

We define this mechanism as the horizontal On–Off response, where HCs analyze the differences between surrounding pixel intensities and a central pixel within a local receptive field [27]. Since the HCs are not able to output to the ganglion cells directly, we designed a specific neuron that presents the cooperation of HCs and other cells like amacrine cells or bipolar cells to emphasize the importance of HCs in direction selectivity [29]. This comparison is made against a predefined threshold θ to activate the horizontal cells. The θ is the minimum resolvable grayscale difference threshold, which is determined based on the dynamic range of human visual perception [56]. A schematic representation of how a local receptive field in a single channel contributes to direction selectivity through horizontal cells is shown in Figure 2b: The aimed central pixel in image *t* is highlighted in red, and the surrounding pixels in image t+Δt are highlighted in blue. All the surrounding pixels are compared with the central pixels by the horizontal cells and influence the final output. For a receptive field, ${\bar{H}}_{\left(a,b\right)}^{c}=\left\{h_{\left(a+y,b+z\right)}^{c},\left(y,z\in \left\{-1,0,1\right\},y^{2}+z^{2}\neq 0,c\in \left\{R,G,B\right\}\right)\right\}$ is the input matrix located at (a,b) for dendritic ganglion cells layer. The output of this kind of specific neuron is

(1) $${\bar{H}}_{\left(a,b\right)}^{c}=\left\{\begin{array}{c} 1,(|x_{\left(a,b\right)}^{c}-x_{{\left(a+y,b+z\right)}^{\prime }}^{c}|<\theta_{H})\\ 0,(|x_{\left(a,b\right)}^{c}-x_{{\left(a+y,b+z\right)}^{\prime }}^{c}|>\theta_{H}) \end{array}\right.\left(y,z\in \left\{-1,0,1\right\},y^{2}+z^{2}\neq 0,c\in \left\{R,G,B\right\}\right),$$

where $\theta_{H}$ is the threshold of the resolvable grayscale value and is set to 3 as the lowest difference that can be recognized.

### 2.3. Dendritic Ganglion Cells in Direction Selectivity

Dendritic neuron models have been shown to be highly valuable in AVS in prior research, including our own previous studies. It has been demonstrated that dendritic-neuron-based visual processing models simulate biological structures by neurons and can effectively process the signals, both spatially and temporally. This relies on the dendritic structure, a fundamental component of neurons that enables nonlinear computations, unlike perceptron-based artificial neural networks, which often overlook these complex interactions [36,37,39]. Compared to multi-layer perceptrons (MLPs), which process input signals through fully connected linear layers followed by point-to-point nonlinearity, our dendritic neuron model introduces localized bio-inspired nonlinear computation. This allows the synaptic and branch structure to modulate the input signals and utilizes the biological structural dendrite, which is ignored in MLPs. Furthermore, unlike MLPs, where all neurons within a layer share the same activation function and global parameters, dendritic neuron models assign unique learnable parameters to each propagation pathway. The set of parameters in each branch allows more localized representations in dendritic neurons to capture the features [57]. Ganglion cells play a crucial role in the visual system as the final stage in the retinal network before relaying visual information to the brain through the optic nerve [58].

In this simulation, the dendritic ganglion cells receive the signals from the HC-equivalent neurons in the retina, as the outputs of bipolar cells are influenced by HC inhibition [24]. Bipolar cells adjust their responses based on input from photoreceptors and horizontal cells, which regulate signal transmission through HC inhibition. When an HC inhibits a bipolar cell, the bipolar cell’s output is suppressed. Thus, the bipolar cell’s response can be regarded as reflecting the inhibitory influence of HCs. The visual system then determines the brain’s final judgment on motion direction detection by utilizing the output from these neurons. These neurons themselves are not inherently direction-selective but contribute to motion processing through their connectivity with direction-selective retinal ganglion cells [32].

**Figure 3:**
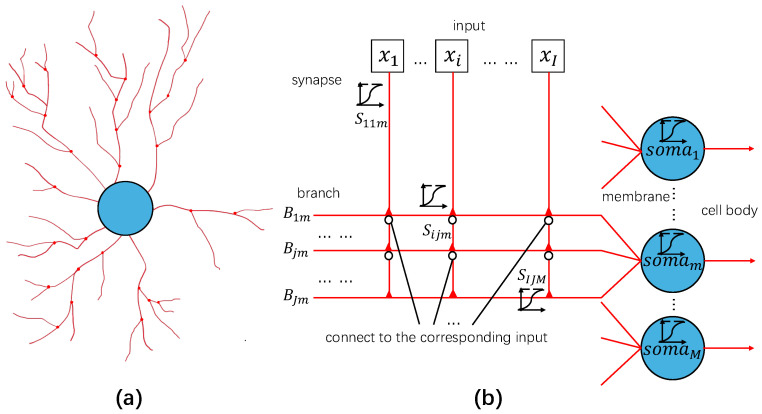
(**a**) is the biological structure image of a neuron with dendritic structure. (**b**) presents the dendritic neuron model structure.

Our dendritic neuron model, in which the ganglion cells consists of four layers, is illustrated in Figure 3. Figure 3a shows the biological structure of a neuron with dendritic structure, while the red strings are the synapses and branches, red circles are the connected nodes, and the blue circle is the cell body with the membrane colored black. Figure 3b presents the dendritic neuron model structure, which includes the inputs, synapse, branch, membrane, and soma layer. The red triangles represent the nodes. The white circle is connected to the input, and since the connection position is not sure, we connect all the inputs with all the branches for every soma. Figure 3b is also a dendritic model that has *I* synapses ($S_{1},\ldots ,S_{i},\ldots ,S_{I}$) or *I* inputs ($x_{1},\ldots ,x_{i},\ldots ,x_{I}$) and *J* branches ($B_{1},\ldots ,B_{j},\ldots ,B_{J}$) and *M* outputs ($o_{1},\ldots ,o_{m},\ldots ,o_{M}$), and it illustrates the process on the *i*th synapse and *j*th branch for a specific output *m*. The sigmoid function is used as the activation function of the dendritic neuron model. The dendrite presents the signal strength from the synapse to the branch, which ends up with the connection to the soma. The sigmoid function enhances the difference near the threshold, which enlarges the exicitatory or inhibitory signal but maintains the gradient, so the whole system is able to learn from the labeled dataset. The process is a follows: 1. The synapse layer receives input signals from the HC-equivalent cells after processing according to the following equation:

(2) $$s_{ijm}\left(x_{i}\right)=\frac{1}{1+exp(-\frac{1}{d_{ijm}}\left(w_{ijm}^{c}x_{i}-q_{ijm}^{c}\right))},$$

where each synapse outputs the corresponding signal $s_{ijm}$. $d_{ijm}$ is the distance parameter, which presents the length of the synapse or the distance from the *i*th input to the *j*th branch of output $O_{m}$. The $w_{ijm}^{c}$ and $q_{ijm}^{c}$ values are the learnable parameters at a specific channel that influence the output of the synapse and finally of the whole system.

2. For the branch layer, each branch $B_{jm}$ processes the synaptic outputs for branch-level outputs $b_{jm}$ using a nonlinear algorithm, which can judge the interaction relationship of incoming signals through the following equation:

(3) $$b_{jm}=\prod_{i=1}^{I}s_{ijm}\left(x_{i}\right).$$

This nonlinear algorithm enables the system to adjust its learning outcomes based on the connection state. The sigmoid function ensures that the output remains close to 0 when the corresponding synaptic connection has minimal influence from the corresponding branch on the decision-making process in the soma. Conversely, it maintains a constant value of 1 when the synaptic inputs have little effect on the branch. This behavior depends on the specific relationship between the parameters $w_{ijm}^{c}$, $q_{ijm}^{c}$, and 0, which are given by the learning process.

3. The membrane layer plays a crucial role in integrating the outputs from the branch layer. Each branch contributes to the membrane through a weighted sum, where the weight $v_{jm}$ determines the influence of the *j*th branch. This process is mathematically represented as

(4) $$u_{m}=\sum_{j=1}^{J}v_{jm}b_{jm},$$

where $b_{jm}$ denotes the output of the *j*th branch, and $v_{jm}$ acts as the weight that scales the contribution of each branch to the overall membrane layer output. This weighted integration ensures that the membrane layer effectively combines information from multiple branches, forming a unified input for the next stage of processing.

4. The soma layer or the cell body layer serves as the final processing stage, where the membrane output is transformed into the dendritic neuron’s output through the activation function. This uses the same activation function with other layers, which introduces a threshold $\theta_{m}$ and a slope parameter $\lambda_{m}$. The output $o_{m}$ is computed as

(5) $$o_{m}=\frac{1}{1+\exp(-\lambda_{m}\left(u_{m}-\theta_{m}\right))},$$

In this equation, $\theta_{m}$ represents the threshold that the membrane potential must exceed to activate the neuron, while $\lambda_{m}$ ($\lambda_{m}>0$) controls the steepness of the sigmoid curve, determining the sharpness of the activation. Together, these parameters allow the soma layer to generate a smooth, continuous output that reflects the neuron’s response to the integrated input.

Particularly in the branch layer, our model implements nonlinear computations that distinguish it from CNNs. This mechanism allows each input to contribute differently based on learned dependencies, more similar to ViT architectures, which capture relationships between different spatial regions.

Furthermore, the use of a sigmoid activation function enhances the stability of the learning process. The model can dynamically adjust synaptic connections during training, enabling it to learn inhibitory, excitatory, and constant response states. This adaptability is particularly important for direction-selective processing, where the model needs to determine relevant features from dynamic visual inputs.

### 2.4. Application of the Dendritic Model to Horizontal Cell Direction Selectivity

Direction selectivity, as discovered by Hubel and Wiesel, is a fundamental property of the visual system. Research has also indicated that horizontal cells exhibit direction selectivity, meaning they can influence motion perception at early stages of processing.

In our model, the dendritic structure in the ganglion cells processes local receptive fields by dynamically integrating inputs from multiple branches. This allows the model to capture the spatial and directional information of the objects in the images more effectively, mimicking the behavior of the retina. By focusing on local receptive fields, our model ensures that the motion direction detection is biologically plausible. This process considers both artificial neural networks and bio-inspired vision systems.

In this simulation, the inputs, denoted as $\bar{X}=\left\{x_{1},\ldots ,x_{i},\ldots ,x_{I}\right\}$, are processed by the dendritic-neuron-based ganglion cell layer. We define the input matrix $\bar{X}={\left[{\bar{H}}_{\left(a,b\right)}^{R}\;{\bar{H}}_{\left(a,b\right)}^{G}\;{\bar{H}}_{\left(a,b\right)}^{B}\right]}^{T}$ to represent a single receptive field for motion direction detection (in this scenario, I=24). This approach enables the model to handle dynamic visual inputs while maintaining the adaptability needed for direction-selective processing.

The synaptic output matrix $\bar{S}=\left[{\bar{S}}_{m}\right]\left({\bar{S}}_{m}=\left[{\bar{S}}_{im}\right]\left({\bar{S}}_{im}={\left[s_{ijm}\right]}^{T}\right)\right)$ is defined with (2) by

(6) $${\bar{S}}_{m}^{\left(a,b\right)}=\sigma \left(\bar{X}\cdot {\bar{\Omega }}_{m}+{\bar{Q}}_{m}\right),$$

where $\bar{\Omega }$ and $\bar{Q}$ are the weight and bias matrix, which are defined as $\bar{\Omega }=\left[{\bar{\Omega }}_{m}\right]\left({\bar{\Omega }}_{m}=\left[w_{ijm}\right]\right)$, $\bar{Q}=\left[{\bar{Q}}_{m}\right]\left({\bar{Q}}_{m}=\left[q_{ijm}\right]\right)$.

In the branch layer, the branch output matrix $\bar{B}=\left[{\bar{B}}_{m}\right]\left({\bar{B}}_{m}={\left[b_{jm}\right]}^{T}\right)$ is defined by

(7) $${\bar{B}}_{m}^{\left(a,b\right)}={\bar{S}}_{1m}^{\left(a,b\right)}⊙{\bar{S}}_{2m}^{\left(a,b\right)}⊙\ldots ⊙{\bar{S}}_{Im}^{\left(a,b\right)},$$

where ⊙ presents the Hadamard product, which represents the elementwise multiplication of corresponding elements in the matrix.

In the membrane layer, the output matrix $\bar{U}=\left[u_{m}\right]$ is

(8) $${\bar{U}}_{m}^{\left(a,b\right)}={\bar{B}}_{m}^{\left(a,b\right)}\cdot {\bar{V}}_{m},$$

where ${\bar{V}}_{m}=\left[v_{jm}\right]$ is the additional weight of the branch.

In the cell body layer, the judgment on motion direction is defined with (5) by

(9) $${\bar{O}}^{\left(a,b\right)}=\sigma \left(\lambda_{m}{\bar{U}}_{m}^{\left(a,b\right)}-\theta_{m}\right),$$

In this scenario, the $o_{m}$ is the computational result of the local receptive field (a,b) in the *c* channel, which results in a local result $o^{{\left(a,b\right)}^{c}}$. For the whole image, the result is calculated by the whole system as an output vector by sumpooling, which is the most closely related processing way as the global detection or other complex behavior that is the most recognized theory in retinal visual systems [59]. The global motion detection follows this theory is defined as

(10) $$\bar{O}=\sum_{a=1}^{H}\sum_{b=1}^{W}{\bar{O}}^{\left(a,b\right)}.$$

This works out a vector $\bar{O}=\left[o_{m}\left(m=1,2,\ldots ,M\right)\right]$ with the probability of the motion direction. As normal artificial neural networks, the output shows the result with maximum probability $\bar{O}$, representing the probability distribution of motion directions.

### 2.5. Learning Algorithm

The learning process in the proposed model is designed to optimize motion direction detection accuracy while ensuring detection efficiency. The training framework consists of supervised learning, gradient-based optimization, and adaptive loss functions, with each contributing to the model’s learning process.

Specifically, supervised learning enables the model to recognize motion features by mapping input stimuli to the corresponding motion directions. Gradient-based optimization is used to fine-tune the synaptic weights matrix $\bar{\Omega }$ and bias matrix $\bar{Q}$ of the dendritic neuron model. Furthermore, the adaptive loss function penalizes incorrect motion direction predictions while reinforcing biologically plausible responses, thereby aligning the learning process with the principles of neural computation.

We also implemented a synaptic pruning mechanism based on the learning process by changing the synaptic parameters, where noncontributing synapses are weakened over time, mimicking biological synaptic plasticity. This ensures that the model remains efficient and adaptive to various motion conditions. By integrating these mechanisms, the proposed model provides a bio-inspired framework for motion detection that CNN-based methods cannot reach in terms of both accuracy and computational efficiency. The direction-selective mechanisms based on retina and dendritic neuron structures facilitates a more biologically faithful simulation of motion perception.

Cross-entropy loss is well suited for classification tasks, as it encourages the predicted probability distribution to closely approximate the true distribution. As a result, the loss function employed in the model is the cross-entropy loss, which is defined as

(11) $$E=-\sum_{m=1}^{8}T_{m}\log\left(\bar{O_{m}}\right),$$

where $T_{m}$ is the correct output for the *m*th direction (the labeled output), and $\bar{O_{m}}$ is the output of the model, which is the predicted probability of direction.

Minimizing the entropy *E* improves the model’s motion direction detection performance. In order to update the weight of the weight matrix $\bar{\Omega }$ and bias matrix $\bar{Q}$ using a backpropagation algorithm, their respective gradients with respect to the loss function *E* must be derived. We assume that the learning rate is η. Using the chain rule, the gradient of *E* with respect to $\Delta \bar{\Omega }=\eta \frac{\partial E}{\partial \bar{\Omega }}$, $\frac{\partial E}{\partial \bar{\Omega }}$ can be worked out as

(12) $$\frac{\partial E}{\partial {\bar{\Omega }}_{m}}=\frac{\partial E}{\partial \bar{O_{m}}}\cdot \frac{\partial \bar{O_{m}}}{\partial {\bar{U}}_{m}}\cdot \frac{\partial {\bar{U}}_{m}}{\partial {\bar{B}}_{m}}\cdot \frac{\partial {\bar{B}}_{m}}{\partial {\bar{S}}_{m}}\cdot \frac{\partial {\bar{S}}_{m}}{\partial {\bar{\Omega }}_{m}}.$$

Similarly, the gradient of *E* with respect to $\Delta \bar{Q}=\eta \frac{\partial E}{\partial \bar{Q}}$, $\frac{\partial E}{\partial \bar{Q}}$ is given by

(13) $$\frac{\partial E}{\partial {\bar{Q}}_{m}}=\frac{\partial E}{\partial \bar{O_{m}}}\cdot \frac{\partial \bar{O_{m}}}{\partial {\bar{U}}_{m}}\cdot \frac{\partial {\bar{U}}_{m}}{\partial {\bar{B}}_{m}}\cdot \frac{\partial {\bar{B}}_{m}}{\partial {\bar{S}}_{m}}\cdot \frac{\partial {\bar{S}}_{m}}{\partial {\bar{Q}}_{m}}.$$

According to (6)–(9), the equation can be simplified as

(14) $$\frac{\partial E}{\partial {\bar{\Omega }}_{m}}=-\sum_{a=1}^{H}\sum_{b=1}^{W}\frac{T_{m}}{\bar{O_{m}^{\left(a,b\right)}}}\cdot \sigma^{\prime }\left(\lambda_{m}{\bar{U}}_{m}^{\left(a,b\right)}-\theta_{m}\right)\cdot \lambda_{m}\cdot {\bar{V}}_{m}\cdot \left(\prod_{k\neq i}{\bar{S}}_{km}^{\left(a,b\right)}\right)\cdot \sigma^{\prime }\left(\bar{X}\cdot {\bar{\Omega }}_{m}+{\bar{Q}}_{m}\right)\cdot {\bar{X}}^{T}$$

(15) $$\frac{\partial E}{\partial {\bar{Q}}_{m}}=-\sum_{a=1}^{H}\sum_{b=1}^{W}\frac{T_{m}}{\bar{O_{m}^{\left(a,b\right)}}}\cdot \sigma^{\prime }\left(\lambda_{m}{\bar{U}}_{m}^{\left(a,b\right)}-\theta_{m}\right)\cdot \lambda_{m}\cdot {\bar{V}}_{m}\cdot \left(\prod_{k\neq i}{\bar{S}}_{km}^{\left(a,b\right)}\right)\cdot \sigma^{\prime }\left(\bar{X}\cdot {\bar{\Omega }}_{m}+{\bar{Q}}_{m}\right)$$

In Equations (14) and (15), $\sigma^{\prime }$ represents the derivative of the activation function, which is the sigmoid function. These gradients facilitate the weight and bias updates required for the optimization process.

The learned weight and bias help the dendritic neuron model through the updated Equation (9). The summation in Equation (10) ensures that the local motion features extracted at different receptive fields contribute commonly to the final motion direction detection. The process is analogous to biological motion processing mechanisms, where multiple receptive fields integrate local motion information to infer global motion direction.

**Figure 4:**
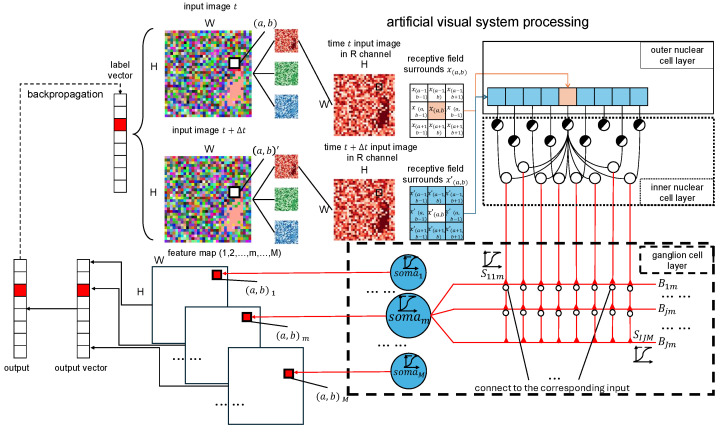
This figure is the overview of the proposed dendritic-neuron-based motion direction detection framework.

The model undergoes iterative training using a gradient-based optimization algorithm, continuously refining weight and bias matrix. Through this learning process, the model effectively captures motion direction information and adapts to different motion direction. The overall framework is illustrated in Figure 4, demonstrating the hierarchical processing structure for motion direction detection tasks. The model processes temporal image sequences, extracting spatial features from receptive fields at different times (*t* and t+Δt). The structure consists of the outer and inner nuclear layers, followed by the ganglion cell layer, which integrates local motion signals. Computations in synapse and branch layers contribute to direction selectivity, leading to a final motion direction detection. The learning process is optimized using backpropagation to refine the weight parameters, as shown by the dashed line arrow.

## 3. Experiment Results

Our experiments were implemented in Python 3.9.16, and Pytorch 2.0.0 was used as the library. The simulations were run on a computer with NVIDIA GeForce RTX 3090.

### 3.1. Dataset Statement

**Figure 5:**
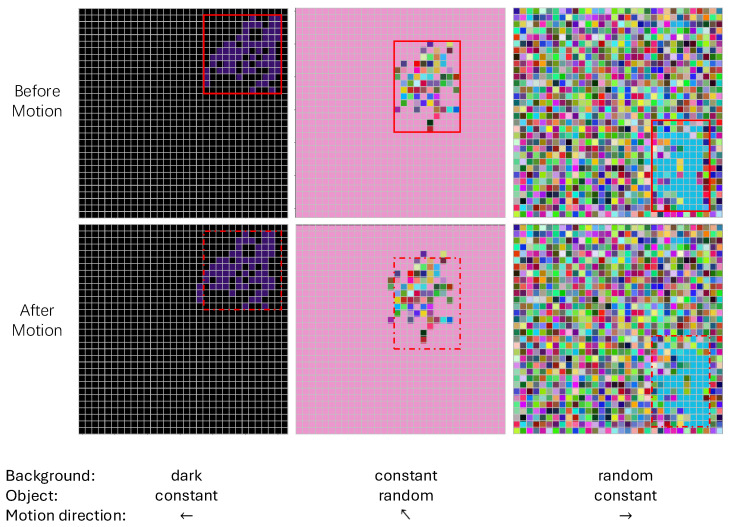
This figure illustrates the dataset samples used for motion direction detection.

In this study, we utilized a custom dataset designed to evaluate the effectiveness of different neuron network models in detecting motion direction across various scenarios, especially our proposed dendritic neuron model. The dataset enables the models to handle multi-channel inputs and perform motion detection in various conditions. The dataset comprises multi-channel (32 × 32 × 3) images that simulate object motion under diverse environmental conditions. These variations are introduced by modifying the RGB channel parameters value of each pixel to represent different lighting and background scenarios. Additionally, the dataset includes objects of varying sizes: 1, 2, 4, 8, 16, 32, 64, and 128 pixels, with each size including 20,000 images. The object shapes are completely random but remain connected, ensuring continuous structures within the images. The dataset is categorized into eight groups based on the background and object settings, which are defined as follows: ‘random’ (all the pixels are given a newly generated random value from 1 to 255); ‘constant’ (all the pixels are given the same generated random value from 1 to 255); and ‘dark’ (all the pixels are given a 0 value). Figure 5 shows the sample images of the dataset. Each row in Figure 5 presents an example before and after motion. The red bounding box highlights the object’s edges before motion, while the red dashed box in the “After Motion” images indicates the previous position for comparison. The motion direction labels and the conditions of the images are shown in the bottom.

To further assess model robustness, we introduced additional noise variations into the dataset. Specifically, salt-and-pepper noise was applied at proportions of 1%, 2%, 5%, and 10% of the total image pixels, with salt (white pixel) and pepper (black pixel) ratios randomized. Additionally, Gaussian noise was incorporated with a mean of 0 and standard deviations of 1, 5, 10, and 20, representing the low, medium, and high noise levels in the experiments under different conditions. These noises allow for evaluating the model’s performance in handling noisy environments, showing the robustness of the models in motion detection tasks.

This simulation dataset helps models to test robustness in real-world applications. The diverse conditions makes it a suitable testing framework for bio-inspired image processing systems.

### 3.2. Bio-Mimetical Experiment on Branch Number

The number of branches directly influences the computational efficiency of the dendritic neuron model by affecting the computational resource demands. However, since our model employs a multiplication-based nonlinear algorithm in the synapse layer, the computational cost increases significantly with the initial number of dendrites, even though the algorithm itself remains relatively simple.

To evaluate the impact of the initial dendritic number, we conducted a series of experiments to perform comparison, assessing the advantages of our model relationship with dendrites. Previous experiments with a bio-inspired model without horizontal cells indicated that the initial dendrite count had a negligible effect on performance. However, as our current model incorporates horizontal cells, this observation may not necessarily hold.

The evaluation considered multiple metrics, including the accuracy and Compute-Normalized Learning Efficiency (CNLE) as *L*. The CNLE provides a fair metric for comparing models that differ in computational resource consumption and is defined as

(16) $$L=\frac{batch\_size\times \Delta E\times \alpha }{R\times P}$$

where batch_size represents the batch size used in training (set as 128 for standardization). ΔE is the improvement in performance over iterations, which is defined by the reduction in cross-entropy loss *E*. α is a coefficient used to ensure that the value of *L* remains within a suitable range ($\alpha =10^{2}$ here). *R* denotes the total training time, which is measured as the duration required to reach a successful training outcome (measured in seconds). *P* represents the GPU utilization percentage, accounting for the computational resources occupied by the model.

This metric ensures fairness when comparing models with different batch sizes and memory requirements, making it particularly suitable for resource-constrained environments. A model with a higher CNLE achieves comparable learning outcomes while consuming fewer computational resources. For the definition of a “successful training” standard in this paper, we first determined the convergence accuracy through extended training epochs. Each model underwent 10 training epochs by suitable epoch, and the final accuracy was averaged. Training was considered successful when the accuracy stabilized within 0.05% of the long-term value, with a standard deviation below 0.1% over 10 epochs to approach the local minimum value. To ensure the training success rate of the model, we selected the maximum epoch count needed from 10 trials, and the runtime required to reach this epoch was used for the CNLE computation. This approach will remain applicable in the subsequent content of this paper.

In this paper, we tested the performance of 1, 5, 10, and 20 initial dendrites. We randomly selected 1250 images from each of the eight datasets, totaling 10,000 images. Among them, 7500 images were used for training and 2500 for testing. The model performance of different initial dendrite branch numbers is shown in Table 1. To distinguish it from our previous model, we used the HCdM (horizontal cell-based dendritic neuron model AVS) to represent the proposed model in the Section 3. In this section, all numerical values in the tables follow the same format. The accuracy values are expressed as mean values with four significant digits, which are accompanied by their standard deviation rounded to three significant digits. The CNLE values are reported with three digits after the decimal point to ensure comparability across models and configurations.

The experiment shows that while increasing the number of dendritic branches has influence little on the accuracy, it does influence the computational efficiency. The CNLE values increased from one to five branches, indicating improved efficiency, but dropped dramatically as the branch number increased further. This suggests that a moderate number of branches balances accuracy and efficiency, whereas excessive branches introduce computational overhead without substantial accuracy gains. Additionally, the HCdM with a higher branch number showed more stable convergence. Since the CNLE values for training and testing remained close values, evaluating training the CNLE alone is sufficient when applying the HCdM.

To evaluate the model performance comprehensively, we conducted experiments across all eight datasets. Cross-validation among the dataset was further applied to assess the model’s robustness and CNLE across different initial dendrite numbers. The cross-validation result is shown in Table 2.

When both the background and object colors were assigned ‘random’, the number of motion features in a single image was significantly higher. This allowed the model to detect motion patterns more quickly, but at the cost of reduced generalization, leading to lower cross-accuracy. Conversely, when the features were limited, such as in the “Constant–Constant” setting, the model detected motion less efficiently but captured more details, resulting in higher cross-accuracy. Additionally, as the branch number increased, the cross-accuracy did not always improve consistently. In some cases, such as in the “Random–Random” condition, the cross-accuracy first decreased and then slightly rebounded at 20 branches. This suggests that while increasing dendritic branches can enhance feature extraction within a dataset, excessive branches may lead to overfitting, limiting the model’s ability to generalize across different datasets. Furthermore, the CNLE values generally decreased with more branches, indicating more stable convergence. However, the stability that more branches brings cannot be ignored.

Additionally, to evaluate the robustness of models with different branch numbers, we conducted noise resistance tests. These tests involved adding salt-and-pepper noise and Gaussian noise to our dataset and assessing the model’s performance under these conditions. The noise immunity test result is shown in Table 3.

The experimental results indicate that the number of branches has little impact on model robustness. After undergoing synaptic-learning-based pruning in the HCdM, the dendritic structures exhibited similar patterns, leading to close performance across different branch numbers. Notably, the model demonstrated strong robustness, particularly against salt-and-pepper noise. Though salt-and-pepper noise over 5% had already surpassed the size of more than half of the objects in our dataset, the model still maintained a high accuracy. Additionally, when dealing with Gaussian noise, the HCdM maintained high accuracy as long as the standard deviation remained around or below the human color discrimination threshold, which is a difference of approximately 3 in RGB values. However, when the noise level significantly exceeded this threshold, its impact on the model’s performance became more pronounced.

### 3.3. Comparison Models

For the task of detecting the motion direction in consecutive frames, it is essential to select models that can efficiently process multi-channel, sequential image data while capturing both spatial and temporal relationships. The state-of-the-art (SOTA) models chosen for comparison here include two kind of tailored ViTs—a dual-stream ViT and spatiotemporal ViT—along with CNN models: EfficientNet, ResNet, and ConvNeXt. Below is a detailed explanation of each model and its role in the context of detecting motion direction.

**The dual-stream vision transformer (2sViT)** processes frames through dual parallel streams with shared ViT weights, where spatial features are independently extracted from each frame for motion detection. While parameter sharing reduces computational overhead for small datasets, this approach needs more resource than CNNs and exhibits limited performance without data augmentation. The 2sViT excels in isolating the spatial features of each frame and then learning the temporal dynamics by combining these features. This separation helps the model focus on specific characteristics in each frame while also understanding how the objects move between them.

**The spatiotemporal vision transformer (stViT)** integrates spatial and temporal information in an integrated pipeline by processing both frames as a single input. Unlike dual-stream approaches, the stViT encodes motion dynamics through self-attention, capturing more motion detection features across frames. To further enhance temporal modeling, we employed long short-term memory (LSTM) between frame representations for spatiotemporal features. This modification improves the motion direction detection by better preserving sequential dependencies compared to traditional stViT architectures. This model is particularly useful for tasks where motion needs to be understood in the context of how it evolves over time.

**EfficientNet-B0 (EfNB0)** is a CNN that optimizes model depth, width, and resolution through compound scaling, achieving high computational efficiency without the accuracy decreasing. It is especially effective for large batch processing, making it suitable for motion detection tasks, since it can quickly process large volumes of data, making it an effective model for detecting movement in consecutive frames while managing computational costs. EfficientNet employs a depthwise separable convolution approach, which reduces the number of parameters and computational complexity while maintaining accuracy.

**ResNet50** is a well-established CNN structure that utilizes residual connections to alleviate the vanishing gradient problem, enabling the training of deep networks. These connections help the network learn complex features without performance degradation as the depth increases. ResNet50 was chosen for motion direction detection because of its balance between resource consumption and accuracy compared to other ResNet models in preprocessing. The depth of ResNet50 allows the model to extract detailed features from both individual frames and their differences. This capability is essential for capturing spatial and temporal patterns critical for detecting motion between consecutive frames.

**ConvNeXt** is a modern convolutional model that combines the strengths of traditional convolutional neural networks with the advancements of transformer-based architectures. The ConvNeXt model is well suited for processing large volumes of multi-channel data, such as consecutive image frames. Its ability to efficiently process both spatial and temporal features makes it effective for motion detection, as it can analyze both overall movement and local changes within frames. Based on preprocessing, we selected the ConvNeXt tiny model for its optimal balance between efficiency and performance.

**The AVS** is based on dendritic neurons and is a specialized model for motion direction detection designed by the authors. It mimics biological visual pathways that include retinal structure and LGN cells, and it is optimized in the HCdM and for synaptic learning. This structure enhances the AVS’s ability to process motion direction efficiently while maintaining biological plausibility.

Each model has a distinct advantage depending on the computational constraints and the complexity of the motion patterns that need to be detected. Combining these models with a dataset designed to simulate object motion under various conditions allows for robust testing and comparison of their ability to handle multi-modal image sequences effectively. To ensure the fairness and reproducibility in the simulations across all models, we followed the same training schedule used in the Section 3.2. Specifically, each model was trained on 7500 images and tested on 2500 images randomly chosen from the dataset. The simulation scoring was based on the accuracy and CNLE in order to enable direct performance comparison. To ensure fairness in model learning, we used the same batch size of 128 and learning rate of 0.001 settings for all models. This batch size was selected to adapt the large-scale models like ViT and ConvNeXt. While our HCdM model is capable of training with much larger batch sizes due to its lower computational demand, we maintained this level to preserve experimental consistency. To further reduce the overfitting risk, we applied L2 regularization to ConvNeXt, particularly in simulations where the model failed to be stable. This adjustment highlights the structural limitations of ConvNeXt in handling aiming motion detection tasks. As CNLE-based efficiency has been clearly analyzed in the Section 3.2, the HCdM efficiency was shown to vary by branch count while remaining high accuracy. Readers interested in the detailed performance tradeoffs of the HCdM are referred to that section. Similarly, the cross-validation results for the AVS model have already been published in previous work using the same evaluation methodology [44].

Since CNNs sometimes struggle with multi-modal images like two frames, we used image concatenation. Although optical flow and difference methods capture some motion features, their detection performance is still inferior to that of handling concatenated images in preliminary experiments. Particularly, we evaluated not only the sparse optical flow method but also implemented dense methods such as the Kanade–Lucas–Tomasi tracking method and the Horn–Schunck method. These flow maps were fed into CNN-based models for comparison. Although such preprocessing is theoretically capable of capturing motion, the experimental results were lower than through image concatenation in most cases. This suggests that such optical flow methods fail to provide reliable features in specific motion direction detection scenario. Image concatenation effectively captures multi-modal information, thus improving the accuracy of motion detection. The training results of the SOTA models are shown in Table 4.

The experimental results highlight the limitations of existing SOTA models in motion direction detection. While all models achieved near-perfect training accuracy results, their test accuracy results dropped significantly, indicating overfitting. This was particularly evident in transformer-based models such as the 2sViT and stViT, although they have a specific structural design for this scenario. The CNNs had higher accuracy results than the ViT models, but the CNLE values indicate that the CNNs’ decision confidence was not consistently reliable. Compared to these models, the HCdM overcomes these limitations by utilizing dendritic-neuron-based processing to capture motion direction features both locally and globally. Unlike SOTA models that overfit or are unable to capture spatiotemporal features, the HCdM is designed to process the continual frame images, ensuring high accuracy and stable performance across training and test datasets. This highlights its superiority in motion direction detection over present models. Since the SOTA models had much lower CNLE values, the comparison with HCdM is meaningless, so the following SOTA model comparison experiment will ignore this coefficient.

To assess the robustness and performance of our model, we conducted several key experiments, including cross-validation and noise immunity. Each experiment was designed to evaluate a specific aspect of model performance under various conditions, ensuring a comprehensive understanding of how well the model generalizes and performs in various scenarios.

#### 3.3.1. Cross-Validation

Cross-validation serves as a crucial technique for evaluating the generalization ability of our model across different visual settings. In these experiments, each neural network was trained on one of the eight different datasets with various color setting and then tested on the mixed dataset. This methodology allows for an in-depth analysis of how the model performs in various object–background combinations, such as light, dark, or random contrasts. By rotating the training and testing sets, we ensured that the model’s robustness and adaptability to unseen visual conditions were thoroughly tested. This simulates some scenarios, where inputs can vary widely and be limited in single position, and highlights the biological realism of our model by simulating the diverse nature of visual inputs. The experimental results are shown in Table 5.

The specialized ViT models, particularly the stViT, performed well in simple settings where both the background and object colors were unified, achieving high test accuracy. However, when more complex scenarios involving random colors were occupied, their accuracy results decreased sharply. In some cases, such as when both the background and object colors were random, the stViT exhibited almost no learning ability, with the accuracy close to random sampling. This suggests that while the stViT can learn well in constrained conditions, it lacks the robustness to handle diverse real-world scenarios. The 2sViT showed more stable performance across different settings but kept a low accuracy, and its overall effectiveness in motion direction detection remained limited. Although the ViT models showed strong performance on the natural image dataset within large scales, their accuracy results dropped dramatically under dark background conditions in our experiments. This phenomenon can be attributed to be the global attention mechanism among the patches. This mechanism relies heavily on the contrast across the entire image and ends up with attention maps that focus narrowly on the patches around the object’s location, making it difficult to recognize spatial–temporal displacement between frames that contribute to motion direction features in dark background images. Additionally, the patch-based input structure of the ViT weakens localized motion information when visual contrast is low, limiting its ability to capture pixel-level features.

Among the CNN-based models, EfficientNet-B0 demonstrated the strongest performance, maintaining relatively high accuracy under controlled conditions, though it was sensitive to environmental randomness. The other CNN models, such as ResNet50 and ConvNeXt, exhibited poor generalization, with their cross-validation accuracy results remaining notably low.

In contrast, the HCdM demonstrated significantly stronger generalization ability. Unlike the SOTA models, the HCdM effectively captured motion direction features across varying conditions. This highlights its superior ability to extract relevant spatiotemporal patterns, making it a more robust and practical solution for real-world motion direction detection tasks.

#### 3.3.2. Noise Immunity

The noise immunity test evaluates the model’s ability to handle noisy or corrupted inputs, which is essential for ensuring its reliability in various scenario. By introducing different levels of noise to the dataset, we tested the model performance under these conditions. We assessed the model’s ability to maintain accuracy and robustness despite disturbances. Since the data quality may vary, the model must remain effective under these conditions to show their robustness. The noise immunity of SOTA model experiment is shown in Table 6.

In this group of experiments, the results show that the SOTA models performed poorly under these noisy conditions. This can be attributed to their complex structures’ focus on capturing global features, which ignores the local motion direction features. The EfN showed strong robustness, as its accuracy remained much more stable than other models despite increasing noise levels. However, its overall accuracy in this setting was already too low, making it unreliable for motion direction detection. The poor performance of specialized ViT models (2sViT and stViT) was unexpected, since their patch-based processing should provide local features, which is unsatisfactory. However, it is worth nothing that the ViT-based models exhibited relatively strong robustness against Gaussian noise, which is considerable.

Compared to traditional models, the AVS showed higher robustness against salt-and-pepper noise but struggled in the high-noise-rate scenario. Its performance dropped sharply as the noise levels increased, indicating its limited robustness. Furthermore, the AVS performed notably worse than the HCdM against Gaussian noise. The possible explanation is probably that bipolar cells introduce unnecessary information that leads to misjudgments in motion direction.

In contrast, our HCdM, through its synaptic learning mechanism on unnecessary connections pruning, remained effective even in high-noise environments. This mechanism helps it to detect motion direction without being overly sensitive to global disturbances. The results particularly highlight the HCdM’s superiority against salt-and-pepper noise, though it was relatively weak on Gaussian noise. The experimental results demonstrate the convinced robustness of the HCdM.

### 3.4. Analysis

The experimental results illustrate a truth that the HCdM remains robust performance across varying branch numbers and datasets. Specially, its synapse pruning, which is based on the learning processing, enables the model to build efficient and approximate dendrite structure regardless of the initial complexity. Compared to SOTA models such as ViTs and CNNs, the HCdM offers significant merits.

While the ViTs struggled with localized feature capturing when facing low-contrast or noisey image, the CNNs tended to overfit and offered limited generalization, and the AVS performed poorly when facing specific noise types and lacks biological theory, the bio-inspired structure of the HCdM ensured stable convergence, high accuracy, and low computational demand. Its localized receptive fields are able to integrate the motion direction features even in challenging environments, which helps to replicate the biological recognition.

Even under severe noise conditions, the HCdM retained impressive accuracy even for salt-and-pepper noise levels that exceeded the object size. These experimental results emphasis its robustness, resource efficiency, and adaptability across datasets.

These findings suggest that the HCdM not only provides biological reasonability but also achieves superior generalization and robustness, making it a convincing direction for bio-inspired motion direction detection models an even useful in real-world applications.

## 4. Discussion

The results of the cross-validation and noise immunity test show that the HCdM demonstrates strong robustness. This suggests that the bio-inspired mechanism, especially its synaptic learning, enable the model to fight against salt-and-pepper noise and Gaussian noise—which is the most noisey condition we meet in real-world environments. However, an interesting finding is that the HCdM exhibited a more significant drop in accuracy under Gaussian noise than salt-and-pepper noise, which is not the case with human visual perception. An unclear mechanism in retinal structure connected to the biological research is probably the reason. An improvement in the future could involve integrating algorithms from ViT-based structures or EfNs to introduce additional global filtering mechanism and learning strategies, since they show comparable stability for this kind of noise.

Another important point is that compared to another bio-inspired model—the AVS—the HCdM does not utilize the LGN structure, which is poorly explained in neuroscience research. Despite this, the HCdM successfully detects motion direction without relying on LGN-based processing, suggesting that the role of the LGN in motion detection might not be as critical as traditionally assumed. However, without direct biological evidence based on experiments, it remains a theoretical hypothesis. Additionally, modeling more complex cortical interactions may further enhance the bio-inspired model’s ability to generalize across different visual conditions and reproduce these tasks as precisely as in human vision.

Another aspect worth exploring is the computational efficiency of the HCdM. The efficiency increases as a result of the synaptic connection and dendritic-neuron-based nonlinear algorithm. Furthermore, the number of branches is adjustable to realize stability levels that can be customized, making the HCdM adaptable to different tasks. Future research could explore hybrid structures, where the HCdM could be combined with self-attention blocks or more efficient feature extraction using SOTA models. These improvements make it possible to design a model that can balance plausibility, robustness, and computational efficiency.

When compared with other studies, quantitative comparisons show that HCdM achieved over 99.5% test accuracy and over 80% cross-accuracy, which are higher than SOTA models, both ViTs (max 50.1%) and CNNs (max 61.7%), under the same experimental settings. Moreover, the HCdM retained its accuracy results above 60% even under 10% salt-and-pepper noise that influenced the integrity of the image, whereas the ViTs and CNNs dropped to around 20%. This confirms the HCdM’s robustness and generalization performance in quantitative terms.

In terms of general applicability, the HCdM is theoretically suitable for motion direction detection tasks, especially where pixel-level motion happens, such as detecting micro-movements in medical imaging or slight displacements in mechanical part monitoring. The ability to extract motion features with low-level computation and the ability to process multi-channel data make the HCdM generalizable. Since the HCdM is better suited to short-term motion detection tasks with specific direction, it is limited with respect to finishing tasks that rely on frame-to-frame displacement vectors compared to optical flow-based methods. In terms of speed, it has low computational requirements and thus runs faster on limited hardware than ViTs or CNNs.

One limitation of the HCdM is that although the HCdM reduces computational costs, the memory footprint may still increase if the branch number is excessively high. This is because each dendritic branch maintains independent parameters. Hence, it is necessary to have a balance between stability and memory efficiency before the total experiments. Additionally, while the HCdM runs efficiently on small-to-medium datasets, it may require structural optimization to scale to large datasets like ImageNet or long video sequences. These limitations should be addressed when deploying the HCdM in large-scale systems. To enhance real-world adaptability, available tools include hybrid modeling like combining the HCdM with CNN layers or ViT-like attention modules or more suitable hardware for dendritic architectures using neuromorphic chips. Moreover, dynamic pruning algorithms and reinforcement learning can be integrated to adaptively adjust the branch structure in response to input conditions.

## 5. Conclusions

This paper introduces a bio-inspired motion direction detection model, demonstrating its effective ability on feature extraction. This model also shows high accuracy and robustness. Through its dendritic processing and synaptic learning mechanisms, the HCdM efficiently simulates the activations of biological neurons, enabling reliable motion direction detection under various conditions. Furthermore, the experimental result shows that it incurs a lower computational cost compared to other deep learning models. This is a signal that the HCdM is a promising alternative to traditional deep learning models in specific scenarios. A remarkable advantage of the HCdM is its adaptive structure, where the number of dendritic branches can be adjusted to balance accuracy and stability depending on the detection task. This provides a high degree of customization for different applications. Although the HCdM achieved strong robustness and accuracy in our experiments, certain limitations present opportunities for future improvements. The current design is optimized for simplified visual scenarios, meaning its performance in complex, dynamic environments like high-intensity Gaussian noise, which represents potential real-world challenges such as low-opacity object occlusions or signal interference, requires further study. Particularly, the simple structure at present is optimized for specific motion direction detection tasks that have short sequences and compare simple images, so the HCdM’s applicability in higher-dimensional images or larger-scaled motion remains to be explored. Enhancing its adaptability to real-world scenarios would improve its practical utilization in auto driving navigation, medical analysis, or other applications.

Since the HCdM showed weaker performance under Gaussian noise than other conditions, future directions may include hybridizing the HCdM with ViT-based attention algorithms or CNNs for their feature extraction abilities. Finally, given the HCdM’s high robustness, its potential applications in real-time motion perception systems merit further investigation, particularly in scenarios where computational efficiency and biological reasonability are essential.

## Figures and Tables

**Table 1:** The performance and efficiency comparison of different branch numbers of HCdM.

Branch Number	Train Accuracy	Test Accuracy	Train CNLE	Test CNLE
1	99.51±0.13%	99.40±0.13%	1.106	1.110
5	99.66±0.26%	99.59±0.36%	1.181	1.182
10	99.55±0.20%	99.53±0.23%	0.236	0.236
20	99.65±0.17%	99.49±0.17%	0.255	0.256

**Table 2:** The cross validation result of HCdM.

Background Color	Object Color	Branch Number	Train Accuracy	Test Accuracy	Cross-Accuracy *	CNLE
Dark	Constant	1	99.44±0.37%	99.42±0.43%	68.05±0.69%	0.064
		5	99.66±0.42%	99.81±0.20%	73.72±7.30%	0.424
		10	99.57±0.72%	99.57±0.66%	76.74±7.63%	0.008
		20	99.57±0.66%	99.48±0.62%	79.06±6.62%	0.007
	Random	1	99.97±0.01%	99.99±0.01%	81.41±4.97%	3.746
		5	99.99±0.01%	99.96±0.04%	81.44±4.29%	0.792
		10	99.90±0.29%	99.86±0.35%	81.51±3.90%	0.151
		20	99.98±0.03%	99.99±0.02%	81.72±3.93%	0.108
Constant	Dark	1	98.73±0.37%	99.14±0.55%	81.34±4.66%	7.251
		5	99.81±0.13%	99.76±0.17%	75.11±4.66%	2.270
		10	99.94±0.03%	99.86±0.07%	80.91±4.34%	0.800
		20	99.46±0.32%	99.29±0.51%	70.18±3.66%	0.307
	Constant	1	98.90±0.28%	99.17±0.32%	94.43±5.91%	1.041
		5	99.20±0.13%	99.08±0.35%	96.33±3.40%	0.419
		10	99.40±0.16%	99.54±0.21%	92.40±4.71%	0.245
		20	99.68±0.21%	99.64±0.32%	93.56±2.91%	0.107
	Random	1	100.0±0.00%	100.0±0.00%	94.03±2.35%	3.269
		5	100.0±0.00%	100.0±0.00%	71.22±3.86%	0.934
		10	100.0±0.00%	100.0±0.00%	74.00±7.09%	0.307
		20	100.0±0.00%	99.99±0.02%	78.66±8.97%	0.122
Random	Dark	1	100.0±0.00%	100.0±0.00%	85.84±1.22%	3.098
		5	100.0±0.00%	100.0±0.00%	86.86±2.96%	8.289
		10	100.0±0.00%	100.0±0.00%	87.58±2.24%	2.957
		20	100.0±0.00%	100.0±0.00%	84.68±5.62%	0.890
	Constant	1	100.0±0.00%	100.0±0.00%	84.55±1.62%	1.713
		5	99.96±0.03%	99.97±0.02%	81.66±2.14%	0.331
		10	100.0±0.00%	100.0±0.00%	75.05±2.11%	0.117
		20	99.98±0.02%	99.98±0.02%	75.45±2.01%	0.039
	Random	1	100.0±0.00%	100.0±0.00%	68.26±8.42%	4.461
		5	100.0±0.00%	100.0±0.00%	48.94±9.22%	9.943
		10	100.0±0.00%	100.0±0.00%	46.84±7.50%	4.330
		20	100.0±0.00%	100.0±0.00%	53.85±9.99%	1.410

* Cross-accuracy represents the accuracy when a model trained on a single dataset is tested across all datasets.

**Table 3:** The noise immunity test of HCdM with different branch numbers.

**Branch Number**	**Salt-and-Pepper Noise Rate**			
	**1**	**2**	**5**	**10**
1	93.48±1.80%	88.98±4.14%	76.52±6.43%	63.26±6.60%
5	93.95±1.79%	89.05±3.93%	77.29±6.29%	64.10±6.52%
10	92.17±2.57%	86.01±4.94%	72.93±5.75%	59.98±5.25%
20	94.54±3.13%	87.19±0.52%	73.91±7.39%	60.90±0.69%
**Branch Number**	**Gaussian Noise Standard**			
	**1**	**5**	**10**	**20**
1	92.32±2.16%	63.08±6.34%	32.03±3.22%	22.28±2.22%
5	90.78±3.91%	59.20±8.06%	31.38±4.52%	22.12±2.48%
10	91.50±3.00%	60.68±5.47%	30.62±3.13%	21.90±1.83%
20	90.68±4.57%	62.32±6.52%	32.45±4.37%	22.29±2.60%

**Table 4:** The performance and efficiency comparison of different SOTA models.

Model	Train Accuracy	Test Accuracy	Train CNLE	Test CNLE
2sViT	100.0±0.00%	38.36±1.10%	0.512	−1.077
stViT	100.0±0.00%	50.06±7.08%	0.052	−0.042
EfNB0	99.97±0.23%	61.67±1.38%	0.021	0.001
ResNet50	97.40±2.50%	19.26±2.40%	0.186	1.556
ConvNeXt	99.80±0.23%	21.53±7.66%	0.001	−0.016

**Table 5:** The cross-validation result of SOTA model.

Background Color	Object Color	Model	Train Accuracy	Test Accuracy	Cross-Accuracy *
Dark	Constant	2sViT	99.99±0.00%	40.66±0.96%	17.60±0.67%
		stViT	100.0±0.00%	94.34±1.51%	33.91±2.08%
		EfNB0	100.0±0.00%	99.95±0.05%	34.38±0.02%
		ResNet50	99.99±0.02%	99.48±1.06%	34.28±0.12%
		ConvNeXt	99.98±0.03%	99.92±0.02%	34.18±0.05%
	Random	2sViT	100.0±0.00%	36.62±0.55%	16.60±0.73%
		stViT	100.0±0.00%	96.58±0.68%	35.06±2.16%
		EfNB0	100.0±0.00%	99.95±0.06%	34.45±0.04%
		ResNet50	100.0±0.00%	99.86±0.12%	34.70±0.05%
		ConvNeXt	100.0±0.00%	99.96±0.04%	34.44±0.08%
Constant	Dark	2sViT	100.0±0.00%	39.12±0.89%	15.56±0.72%
		stViT	97.85±3.77%	58.62±10.4%	25.81±4.24%
		EfNB0	98.68±0.05%	99.53±0.12%	59.27±4.86%
		ResNet50	99.66±0.06%	37.41±6.98%	18.78±1.68%
		ConvNeXt	99.97±0.06%	99.55±0.13%	39.31±0.18%
	Constant	2sViT	99.97±0.04%	37.60±1.14%	16.93±0.88%
		stViT	99.98±0.03%	36.58±1.34%	15.93±0.69%
		EfNB0	100.0±0.00%	99.56±0.14%	66.72±0.04%
		ResNet50	100.0±0.00%	14.61±1.20%	14.12±0.10%
		ConvNeXt	99.09±0.65%	27.60±18.7%	21.05±10.3%
	Random	2sViT	100.0±0.00%	36.07±0.86%	16.30±0.92%
		stViT	100.0±0.00%	35.81±1.10%	16.07±0.85%
		EfNB0	100.0±0.00%	99.72±0.14%	66.96±0.05%
		ResNet50	99.99±0.02%	21.18±6.57%	16.85±2.32%
		ConvNeXt	99.82±0.20%	94.77±7.01%	43.44±3.87%
Random	Dark	2sViT	100.0±0.00%	37.86±0.96%	16.57±0.65%
		stViT	13.04±0.37%	15.52±0.92%	12.55±0.62%
		EfNB0	100.0±0.00%	24.92±0.33%	14.12±0.05%
		ResNet50	100.0±0.00%	17.03±2.45%	13.11±0.39%
		ConvNeXt	99.98±0.03%	16.81±4.35%	14.58±0.33%
	Constant	2sViT	100.0±0.00%	35.01±1.04%	15.64±0.72%
		stViT	13.14±0.35%	12.84±0.72%	12.65±0.70%
		EfNB0	100.0±0.00%	12.68±0.87%	12.55±0.09%
		ResNet50	100.0±0.00%	12.26±0.51%	12.37±0.08%
		ConvNeXt	99.96±0.07%	12.30±0.90%	12.56±0.04%
	Random	2sViT	99.76±0.76%	35.47±1.06%	15.36±0.70%
		stViT	13.31±0.29%	12.36±0.50%	12.63±0.40%
		EfNB0	100.0±0.00%	12.57±0.72%	12.58±0.08%
		ResNet50	100.0±0.00%	23.39±0.45%	12.60±0.06%
		ConvNeXt	99.74±0.53%	12.86±0.43%	12.41±0.04%

* Cross-accuracy represents the accuracy when a model trained on a single dataset is tested across all datasets.

**Table 6:** The noise immunity test of different SOTA models.

**Model**	**Salt-and-Pepper Noise Rate**			
	**1**	**2**	**5**	**10**
AVS	89.69±3.28%	82.09±4.22%	68.86±4.25%	57.64±3.56%
2sViT	16.93±0.98%	15.89±0.46%	14.49±1.00%	13.52±0.47%
stViT	22.09±2.55%	19.35±1.49%	16.57±0.62%	14.75±1.01%
EfN	25.17±1.21%	21.08±0.93%	17.83±0.96%	15.36±0.80%
ResNet	17.08±1.27%	15.89±1.05%	14.88±0.88%	13.90±0.73%
ConvNeXt	18.99±3.42%	17.69±3.01%	15.79±1.60%	14.86±1.04%
**Model**	**Gaussian Noise Standard**			
	**1**	**5**	**10**	**20**
AVS	92.16±3.84%	69.33±7.43%	40.62±3.68%	28.03±1.50%
2sViT	18.82±0.72%	18.10±0.84%	18.77±0.73%	17.46±0.94%
stViT	33.40±7.52%	33.04±7.74%	31.70±6.91%	28.90±4.84%
EfN	62.46±2.17%	59.99±2.12%	43.02±2.07%	29.96±2.02%
ResNet	22.10±2.71%	22.64±2.57%	22.15±2.25%	22.01±2.87%
ConvNeXt	20.21±4.61%	20.04±4.29%	18.19±3.09%	16.68±1.88%

## Data Availability

The data used in this study are limited access but available for reasonable requirement. Interested parties may request access by contacting the corresponding author at yktodo@se.kanazawa-u.ac.jp. Access is subject to approval and compliance with confidentiality and ethical guidelines. Simulation result: https://wandb.ai/ruriiiii/HCRGB-Horizontal_Dendrite_Direction (accessed on 25 April 2025). Code and program: https://github.com/RurIIIIIkitesumimasen/HCdM (accessed on 25 April 2025).
